# The Public Servants of the Future

**Published:** 1889-09-21

**Authors:** 


### The Hospital

A Weekly Institutional Journal of

**Science, Medicine, Nursing, and Philanthropy.**

*For Hospitals, Asylums, Medical Practitioners, Students, Nurses, Families, Parishes, Congregations, and the Charitable Public.*

Vol. VI. No. 156.] [September 21, 1889.

### The Public Servants of the Future.

Some of the daily papers have complained that the President of the British Association and his brother presidents of sections have given the public nothing of a new, startling, or extraordinary character at their meeting at Newcastle this year. If there had been anything in the world of science particularly new or marvellous we should have heard of it, no doubt; but the scientific year having been comparatively quiet and uneventful, the summary of it contained in the Newcastle addresses was bound to be quiet and uneventful too. The president's address on museums was for the benefit of specialists primarily, but in the long run will serve the convenience of the public also. The question was by no means a "burning" one, neither was the treatment of it. Mr. Francis Galton dealt with a subject of much greater immediate importance, and one which has vastly wider bearings than were considered at the sectional meeting.

For the public services of this country—the Army, the Navy, and the Indian Civil services more especially—there is a general consensus of opinion that the best men should in every case be selected. Very few persons will venture to question that. But the point is how to get hold of the best men. Everybody knows that since the days of patronage a literary competition has been the sole test of merit. It was thought at first that such a competition would be all-sufficient, and that by its means there would be no difficulty in selecting from any number of candidates the men who were best fitted for the particular duties required of them. But experience has shown, and continues to show, that a literary competition as a test of all-round merit and fitness is a comparative failure. It might, we think, have been foreseen at the outset that such would be the case; and probably it was foreseen by many; but as it was considered that the old patronage system was the worst that could possibly have been devised, so it was held that any method which would give rewards to proved merit was sure to be better than it. We are come now to a time when the necessity for the choice of the best men is seen to be more urgent than ever; and any man who shall discover the best or a better than the existing method of literary competition, for finding them, will deserve not only the thanks of his fellow-countrymen, but the much higher compliment of the prompt acceptance of his views and recommendations.

Mr. Francis Galton, who read a paper before the Anthropological section, takes it for granted that the literary method has been at least a partial failure. It is useful so far as it goes; but it is not sufficient. Mr. Galton proposes, not a substitute for it, but an addition to it, in the shape of what we may call an "athletic" test. We venture to think, however, that even the addition of the athletic test will not give us the best men. In order to satisfy all the demands of the occasion, we must have, as the complement of the intellectual and the physical, a competent "moral" test. The last, which seems to us the most important factor of all, has been entirely left out of the question by Mr. Galton; and, so far as we can discover, by all the other members of the anthropological section who took part in the discussion.

Those who wish the public services to be administered, as all such services should be administered, in the most honest and efficient way, must divest themselves of all preconceived notions of every kind and fix their minds exclusively on two things—viz., the work to be done, and the men who are to do it. Now, the work demands at least three kinds of fitness. First and emphatically there must be physical fitness. For if a man cannot live and retain health under the conditions which the work imposes he cannot do any duties at all, either efficiently or inefficiently. In placing intellectual before physical fitness, therefore, the advocates of a literary test put the cart before the horse. Mr. Francis Galton is clearly on the right tack when he strives, though late in the day, to put the horse before the cart. The logical order of fitness, it is plain as facts can make it, is first physical. Not only should all candidates be examined as at present by medical men in order to ascertain their freedom from actual disqualifying disease or weakness, but they should be competitively tested also according to a standard of physical energy and perfection, and receive marks for merit exactly as in the present intellectual competition.

Of the literary examination it is unnecessary to speak at length. Although it satisfies very few persons, yet it is felt to be better than the old method; and no one would propose to get rid of it until a better test is ready for substitution. There must, in fact, always be a literary examination as one department of the qualifying tests; but that something is required in addition, nobody who knows anything of candidates for the services, of "crammers," and of examinations, will for a moment doubt. It is probable that the vast majority of those who are interested in the subject will consider that the addition of a competition in athletic exercises, with other tests for manifesting varieties of physical capacity, will give the public all that can reasonably be required.

But, recurring to the principle laid down before, that it is above all things necessary to consider the nature of the duties to be performed by our public servants, is it not clear that a certain fitness of sense and moral character is one of the most indispensable qualifications to be looked for? What do we mean by moral character and sense? We do not mean merely that a man must be above the level of lying, stealing, and cheating at cards. That we take for granted every candidate for any of the public services already is. But when a man is selected to be placed over large numbers of his fellow-men, as in a regiment; when he becomes, so to speak, their earthly providence and the arbiter of their destinies, we submit that something more is required than that he should know it to be dishonourable to cheat at cards or to be guilty of a lie. There are wanted, in short, men of open mind, of judicial temper, of a reasonable and sympathetic spirit; men who can put themselves, in imagination, in the place of their subordinates, and mete out to them that measure of noble humanity which consists in doing to inferiors as they would wish to be done by. Still more is this the case where, as in the Indian civil service, men are appointed to be rulers and judges of large numbers of their fellow-creatures who are alien in race, in religion, in education, and in all the manners and customs of their daily lives. Do we want our fellow-subjects in India to be ridden rough-shod over by coarse louts, who, if left to their own devices in this country, would pass from the school to the stable and from the stable to the gutter? Yet that is what has often been done, not only in the civil service of India, but also in the army, and, perhaps, more in the army than in the civil service. Those who know by experience the inside of a "crammer's" establishment can understand very easily how these things may be done. Of all the uncultured barbarians that this country produces, in any rank of life, the young men who are being "coached" by the different crammers for the public services are in actual behaviour probably the most barbarous. In some of them, brutal selfishness and indescribable vulgarity have surely reached the utmost limits of possibility. Yet these are the men we send out to command our troops all the world over, and to rule the millions of our helpless fellow-countrymen in that great dependency which is the pride and glory of every imperial British heart. Let us by all means have physical tests for admission to our public services, and let us have intellectual tests too. But if the chief value of every man in every position be his high moral worth, let us make at least a little effort to give to our soldiers officers whom they can trust and obey, and to our Indian fellow-subjects judges who shall make our rule honoured and loved because it is just and equal. Without moral qualifications the stoutest bodies and the clearest heads will entirely fail to carry out the highest principles of private and public justice, on which alone the stability of this great empire must ultimately depend.

